# Mechanisms of mono- and poly-ubiquitination: Ubiquitination specificity depends on compatibility between the E2 catalytic core and amino acid residues proximal to the lysine

**DOI:** 10.1186/1747-1028-5-19

**Published:** 2010-08-13

**Authors:** Martin Sadowski, Boris Sarcevic

**Affiliations:** 1 Cell Cycle and Cancer Unit, St. Vincent's Institute of Medical Research, St. Vincent's Hospital, University of Melbourne, Fitzroy, Melbourne, Victoria 3065, Australia; 2 Department of Medicine, St. Vincent's Hospital, University of Melbourne, Fitzroy, Melbourne, Victoria 3065, Australia

## Abstract

Ubiquitination involves the attachment of ubiquitin to lysine residues on substrate proteins or itself, which can result in protein monoubiquitination or polyubiquitination. Ubiquitin attachment to different lysine residues can generate diverse substrate-ubiquitin structures, targeting proteins to different fates. The mechanisms of lysine selection are not well understood. Ubiquitination by the largest group of E3 ligases, the RING-family E3 s, is catalyzed through co-operation between the non-catalytic ubiquitin-ligase (E3) and the ubiquitin-conjugating enzyme (E2), where the RING E3 binds the substrate and the E2 catalyzes ubiquitin transfer. Previous studies suggest that ubiquitination sites are selected by E3-mediated positioning of the lysine toward the E2 active site. Ultimately, at a catalytic level, ubiquitination of lysine residues within the substrate or ubiquitin occurs by nucleophilic attack of the lysine residue on the thioester bond linking the E2 catalytic cysteine to ubiquitin. One of the best studied RING E3/E2 complexes is the Skp1/Cul1/F box protein complex, SCF^Cdc4^, and its cognate E2, Cdc34, which target the CDK inhibitor Sic1 for K48-linked polyubiquitination, leading to its proteasomal degradation. Our recent studies of this model system demonstrated that residues surrounding Sic1 lysines or lysine 48 in ubiquitin are critical for ubiquitination. This sequence-dependence is linked to evolutionarily conserved key residues in the catalytic region of Cdc34 and can determine if Sic1 is mono- or poly-ubiquitinated. Our studies indicate that amino acid determinants in the Cdc34 catalytic region and their compatibility to those surrounding acceptor lysine residues play important roles in lysine selection. This may represent a general mechanism in directing the mode of ubiquitination in E2 s.

## Introduction

**Figure 1:**
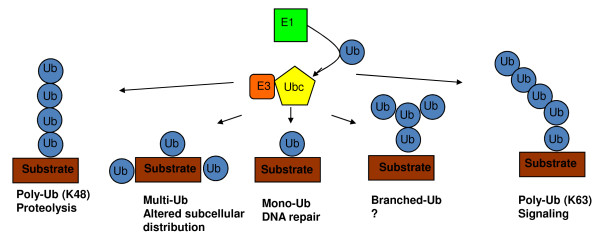
**Different modes of ubiquitination lead to different substrate fates**. The versatility of Ub in regulating different processes is derived from its ability to be conjugated as a monomer on one (monoubiquitination) or more substrate lysines (multiubiquitination) or as a polymer (polyubiquitination) by the sequential addition of further Ubs to each other through Ub lysines. Since Ub contains seven lysines, polyubiquitination can generate linear or branched chains with different topologies. Monoubiquitination can regulate DNA repair, viral budding and gene expression, while polyubiquitination through K48 of Ub generally results in proteasomal degradation, and K63-linked Ub chains can function in signaling and endocytosis.

Ubiquitination is a fundamental biochemical process, which controls numerous aspects of protein function, such as degradation, protein-protein interaction and subcellular localization [1]. The attachment of the 8 kDa protein ubiquitin (Ub) to proteins involves three classes of enzyme, an E1 ubiquitin-activating enzyme, an E2 ubiquitin-conjugating enzyme, and an E3 ubiquitin ligase. The C-terminus of Ub first forms a thioester bond with the catalytic cysteine of the E1 in an ATP-dependent manner. Ub is then transferred from the E1 to the catalytic cysteine of the E2. Finally, the E3 binds both the Ub-charged E2 and substrate to catalyze transfer of the C-terminus of Ub to a substrate lysine to form an isopeptide bond, resulting in substrate monoubiquitination. Substrates can be ubiquitinated on numerous lysines, resulting in multiubiquitination [2,3]. In addition, some E2/E3 combinations can then utilize lysines on the substrate-conjugated ubiquitin, to catalyze further cycles of ubiquitination, resulting in substrate polyubiquitination [1,3]. Ub contains seven lysines, which can be utilized during polyubiquitin chain formation, and in most cases a specific lysine is utilized by a particular E2/E3 pair [3]. The ability to generate diverse substrate-ubiquitin structures is important for targeting proteins to different fates. For example, monoubiquitination can regulate DNA repair and gene expression [4]. Polyubiquitination through Ub K48 generally targets proteins for proteasomal degradation, while K63-linked Ub chains can regulate kinase activation, DNA damage tolerance, signal transduction and endocytosis [4] (Figure 1).

The mechanisms that control lysine selection in substrates are not clearly understood. Structural aspects of E2/E3 s and how they bind the substrate are believed to be important. Therefore, a "positioning model" posits that the E3 positions the substrate toward the E2~Ub thioester bond to select particular lysines during substrate ubiquitination [3,5]. However, some substrates are known to be ubiquitinated on numerous lysines, e.g. the budding yeast CDK inhibitor Sic1 [2]. It is believed that binding of the substrate through several binding motifs to the E3 results in various binding geometries, leading to ubiquitination on numerous lysines. Other studies have suggested that substrate lysine selection flexibility may be achieved by release of the ubiquitin-charged E2~Ub from E3, to transfer Ub to different substrate lysines, as proposed by the'hit and run' model [6]. During polyubiquitin chain extension structural features in E2 s position Ub lysines to generate Ub chains via a specific lysine [7,8]. For example, the Mms2/Ubc13-Ub complex assembles so that K63 of Ub attacks the Ubc13-Ub thioester bond during Ub chain formation [9]. Similarly, an acidic loop region in the E2 Cdc34 is thought to position K48 of Ub for attack of the Cdc34~Ub thioester bond [10]. Other E2 s, such as human UbcH5, can utilize several Ub lysines (K11, K48 and K63), indicating less structural constraint and that other mechanisms may also contribute to Ub lysine selectivity [8].

**Figure 2:**
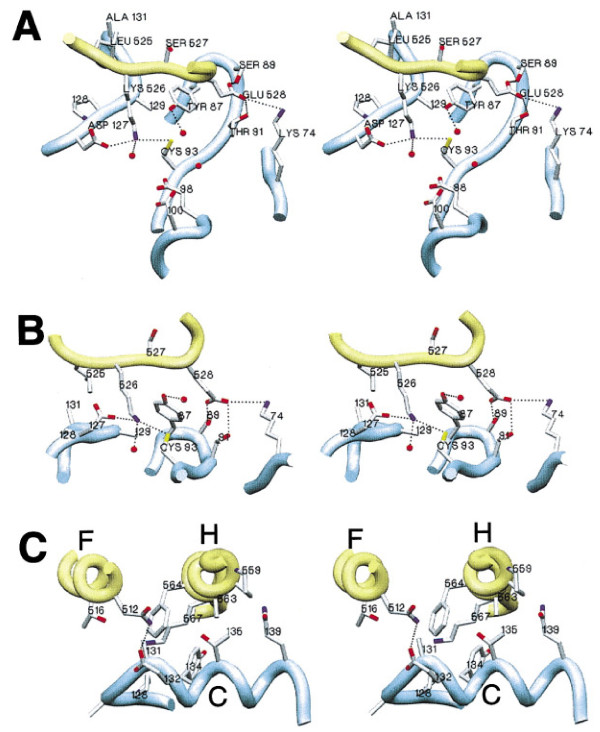
**Stereo Images of Ubc9-RanGAP1 Interaction**. (A and B) Orthogonal orientations of the Ran-GAP1 consensus motif in complex with Ubc9. Amino acids are indicated by type, number, or both. Ubc9 Glu98 and Asp100 are removed in (B). (C) RanGAP1-Ubc9 interaction outside the motif recognition interface. Helices lettered for RanGAP1 (F and H) and Ubc9 (C). Backbonepositions are represented by ribbon spline (RanGAP1 in yellow, Ubc9 in blue), theoretical hydrogen bonds are dotted lines, and waters are red spheres. *Reprinted from Cell, 108 (3), Bernier-Villamor V, Sampson DA, Matunis MJ and Lima, CD, Structural basis for E2-mediated SUMO conjugation revealed by a complex between ubiquitin-conjugating enzyme Ubc9 and RanGAP1, 345-356., Copyright (2002), with permission from Elsevier.

Apart from higher order structures of E2 s and E3 s contributing to lysine selection in substrates and Ub, it is not clear if amino acid determinants within the catalytic region of E2 s and those surrounding substrate and Ub lysines play a role in lysine selectivity. At a catalytic level, ubiquitination of substrate or Ub lysines occurs by nucleophilic attack of the lysine residue on the E2~Ub thioester bond. Recent studies with the human anaphase promoting complex (APC/C) RING E3 and its E2, UbcH10, have identified a sequence motif adjacent to acceptor lysines, termed the TEK-box, which is important for ubiquitination [7], suggesting that amino acid determinants near the lysine residue may play a crucial role in lysine selection. Initially, insights into the importance of amino acids surrounding acceptor lysines and the E2 catalytic core have come from studies of the SUMO-conjugating E2, Ubc9. Ubc9 attaches the ubiquitin-like protein SUMO onto substrate lysines in an analogous fashion to that used by ubiquitin-conjugating E2 s. Structural studies of Ubc9 complexed with its substrate, RanGAP1, show that amino acids in proximity of the catalytic cysteine make important contacts with amino acids surrounding RanGAP1 K526, which is sumoylated. Hence, Ubc9 Y87 and A129 make van der Waals contacts with L525, S527 and E528 of RanGAP1, which are proximal to the sumoylated K526, while Ubc9 D127 is within hydrogen-bonding distance of sumoylated RanGAP1 lysine 526 [11,12] (Figure 2). These interactions facilitate sumoylation through optimal alignment and pK suppression for nucleophilic activation of the attacking RanGAP1 K526 [11,12].

## Discussion

Since previous studies indicate that amino acids in the SUMO E2 catalytic site interact with amino acids surrounding the attacking lysine to help position it for nucleophilic attack [12], we explored if analogous sites contribute to lysine specificity in ubiquitin-conjugating E2 s. We utilized the yeast RING E3 ligase Skp1-Cdc53/cullin-F box protein (SCF) and its cognate E2, Cdc34 to assess ubiquitination of their physiological substrate, Sic1 [3]. In *S. cerevisiae*, G_1_-S phase cell cycle progression depends on SCF^Cdc4^/Cdc34-mediated polyubiquitination of Sic1 via K48-linked Ub chains, leading to its proteasomal degradation [2,13,14].

### Amino acids proximal to Sic1 lysines and Ub lysine 48 are critical for substrate ubiquitination and Ub chain extension by Cdc34/SCF^Cdc4^

Our studies analyzing ubiquitination of six different lysines in the N-terminus of Sic1 (K32, K36, K50, K53, K84, and K88) demonstrated that SCF^Cdc4^/Cdc34 displayed a clear preference toward Sic1 K53 [15]. The importance of the sequence environment was exemplified by our studies mutating residues around Sic1 lysines, where changes to proximal amino acids consistently reduced or increased ubiquitination. We changed the flanking residues of poorly ubiquitinated Sic1 lysines (K32, K84 and K88) to those of efficiently ubiquitinated sites (K50 and K53) and *vice versa*. The rationale was to determine if keeping the lysine position unchanged, but altering its proximal amino acids can alter the level of ubiquitination. These studies showed that substituting amino acids around optimal K53 with those found around poorly ubiquitinated K32 and K84, substantially reduced K53 ubiquitination. Conversely, substituting amino acids around poorly ubiquitinated K32 with those found around optimal K53, significantly increased K32 ubiquitination. Hence, amino acids proximal to lysine residues are critical for controlling their efficiency of ubiquitination. Importantly, the efficiency of ubiquitination of these Sic1 mutants *in vitro*, correlated with their rate of degradation in *S. cerevisiae*, which controlled the growth rate in this organism. Therefore, amino acids proximal to Sic1 lysines play an important role in the rate of degradation of this protein to control cell cycle progression in *S. cerevisiae*, underscoring their physiological importance. In addition, our studies also revealed that amino acids around Ub K48 are important determinants of catalysis during chain extension via Ub K48.

### Amino acids in the catalytic core of Cdc34 specify Sic1 monoubiquitination or polyubiquitination

Importantly, our studies linked this sequence-dependent effect of ubiquitination efficiency to key residues in the catalytic region of Cdc34, which can differentially regulate initial Sic1 substrate ubiquitination and Ub chain extension via K48, and thus specify if Sic1 is monoubiquitinated or polyubiquitinated. This was strikingly exemplified by two Cdc34 mutants (S139D and Y89N), which displayed polar opposite activities toward lysines of Sic1 and K48 of Ub. Cdc34 (S139D) was as active as wild-type Cdc34 in ubiquitination of Sic1 lysines but essentially inactive toward K48 of Ub and thus impaired in polyubiquitination. Conversely, Cdc34 (Y89N) was substantially impaired in Sic1 ubiquitination but active toward K48 of Ub during polyubiquitination. Therefore, these mutants were not inactive *per se*, but rather displayed differential specificity toward particular acceptor lysines. Hence, the Cdc34 (S139D) mutant demonstrated that a single point mutation in the catalytic core can convert Cdc34 from a polyubiquitinating E2 into a monoubiquitinating E2. It is known that some E2 s prefer to monoubiquitinate substrate proteins, while other E2 s only catalyze Ub chain elongation on pre-conjugated substrate-Ub complexes [16]. However, the mechanism for this differential specificity is not understood. Our work now suggests that the catalytic regions of E2 s have evolved different active site structures through divergence of key residues to accommodate specific lysines in substrates and/or ubiquitin and thus may dictate if a substrate is mono- or poly-ubiquitinated. This concept is supported by the 'restricted' amino acid divergence found at these core sites in all E2 s.

### Compatibility between Cdc34 catalytic residues and amino acids proximal to lysine is important for ubiquitination

Our studies with the Cdc34 (S139D) mutant highlighted the importance of compatibility between amino acids surrounding the acceptor lysine and E2 core residues. While this mutant was impaired in polyubiquitination due to its inability to utilize Ub K48, changes to the Ub K48 proximal residues (Q49P and L50S) restored the activity of this mutant in Ub chain assembly. These studies suggest that key amino acids in the E2 active site interact with key amino acids around the acceptor lysine to position it for nucleophilic attack of the thioester bond during catalysis. This is consistent with previous studies of the SUMO E2-substrate complex of human Ubc9 and RanGAP1, which demonstrate that Ubc9 Y87, D127 and A129 surround the catalytic cysteine and make important contacts with residues surrounding the sumoylated K526 [11,12] (Figure 2). These interactions optimally align and activate K526 for nucleophilic attack through pK suppression [12]. Based on our observations, we speculate that sequence and structural divergence in the catalytic regions of E2 s is an important mechanism for generating E2 s with differential lysine specificity.

## Conclusions

Ubiquitin attachment to specific lysine residues in protein substrates and itself is important for generating diverse substrate-ubiquitin structures, providing versatility to this pathway in targeting proteins to different fates. The mechanisms of lysine selection, however, remain unclear. While it has been appreciated that higher order structural features of E2 s and E3 s are important for lysine selection during ubiquitination, our recent studies showed that compatibility between key residues within the E2 catalytic region and those proximal to acceptor lysines in the substrate or Ub provides a further level of specificity control. The interplay of both mechanisms ultimately defines the efficiency of ubiquitination of a lysine. We speculate that E2 s may have evolved divergent structures in their catalytic regions to modulate lysine specificity. This likely contributes to the mechanisms for different E2/E3 combinations generating structural diversity by controlling if a protein is monoubiquitinated, multiubiquitinated or polyubiquitinated. Further biochemical and structural studies are required to define all of the important determinants in the catalytic region of Cdc34 and residues proximal to acceptor lysines to comprehensively define the interaction interface of the Cdc34-substrate complex. In addition, since we propose that this mechanism is generally important for lysine selectivity during ubiquitination, it will be important to characterize other E2 family members for lysine specificity.

## List of Abbreviations

Ub: ubiquitin; E1: ubiquitin-activating enzyme; E2: ubiquitin-conjugating enzyme; E3: ubiquitin ligase; Cdc34: cell division cycle gene 34; RING: really interesting new gene; SCF: Skp1/Cul1/F box protein; SUMO: small ubiquitin-like modifier

## Competing interests

The authors declare that they have no competing interests.

## Authors' contributions

MS and BS drafted, read and approved the manuscript.
